# Transcriptome-wide reprogramming of N^6^-methyladenosine modification by the mouse microbiome

**DOI:** 10.1038/s41422-018-0127-2

**Published:** 2018-12-17

**Authors:** Xiaoyun Wang, Yan Li, Wenjun Chen, Hailing Shi, A. Murat Eren, Aleksey Morozov, Chuan He, Guan-Zheng Luo, Tao Pan

**Affiliations:** 1 Department of Biochemistry and Molecular Biology, The University of Chicago, Chicago, IL 60637 USA; 2 State Key Laboratory of Biocontrol, School of Life Sciences, Sun Yat-sen University, Guangzhou, Guangdong 510275 China; 3 Department of Molecular Genetics and Cell Biology, The University of Chicago, Chicago, IL 60637 USA; 4 Department of Chemistry, The University of Chicago, Chicago, IL 60637 USA; 5 Howard Hughes Medical Institute, The University of Chicago, Chicago, IL 60637 USA; 6 Department of Medicine, The University of Chicago, Chicago, IL 60637 USA; 7 Marine Biological Laboratory, Woods Hole, MA 02543 USA

**Keywords:** Transcriptomics, Epigenetics analysis

Dear Editor,

Microbiome affects many aspects of human health and disease and elicits a wide range of host responses including remarkable epigenetic changes such as DNA methylation, histone modification and non-coding RNA expression.^1^ A still poorly explored area of microbiome-host interaction is the response of host RNA modification. N^6^-methyladenosine (m^6^A) is the most abundant mRNA modification in mammalian cells, occurring at ~3 modified adenosine residues per transcript. The m^6^A mapping and biology have been extensively studied recently.^2^ At the physiological level, m^6^A affects embryonic development, circadian clock, immuno-response, and others. At the cellular and molecular level, m^6^A affects all key aspects of mRNA processing, translation and decay. Importantly, m^6^A is a predominant, transcriptome-wide mark that is responsive to environmental changes; this dynamic m^6^A pattern is maintained by the writer enzyme complex containing the METTL3 and METTL14 proteins, and two eraser enzymes of FTO and ALKBH5.^3,4^

We investigated the host response marked by m^6^A in the transcriptome to the presence of microbiome in mice (Fig. 1a). We employed one group of germ-free (GF) mice to identify the host response to the absence, and the other group of specific pathogen-free (SPF) mice to identify the host response to the presence of microbiome. We validated the absence of gut microbiota in our GF mice by PCR of the representative 16S genes (Supplementary information, Fig. S1a). 16S rRNA gene amplicon sequencing of the SPF mice showed that all three mice in this group had similar bacterial compositions at the genus level, which were mainly blautia and roseburia (Supplementary information, Fig. S1b).

**Fig. 1:**
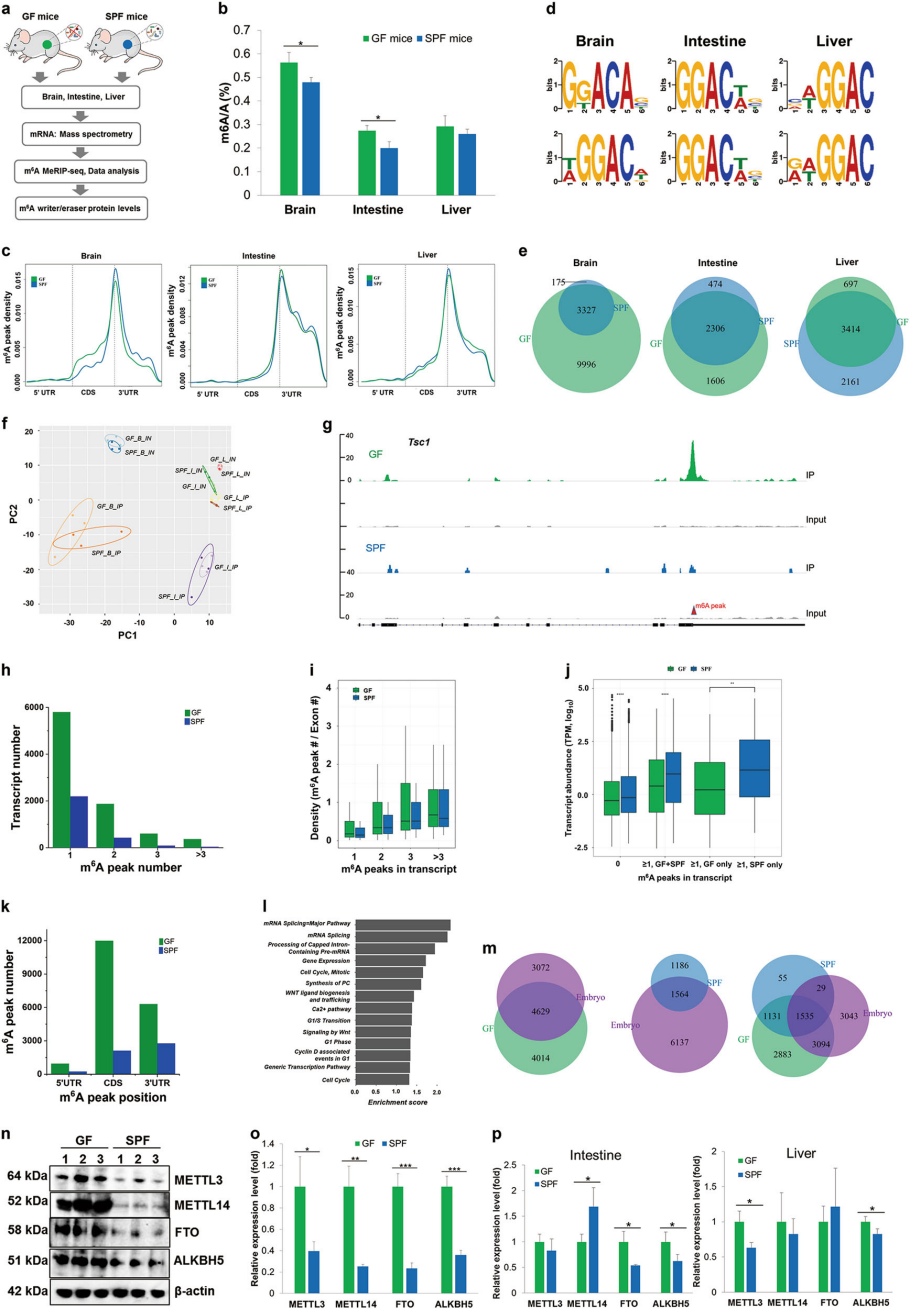
m^6^A methylome and writer/eraser expression in the germ-free (GF) and specific pathogen-free (SPF) mouse tissues. **a** Schematic representation of the study. **b** QQQ LC/MS measurement of total m^6^A/A ratio of polyA-selected and ribo-minus treated RNAs. Values are the means ± standard deviation (SD), *n* = 3, *P < 0.05, Student’s *t*-test. **c** m^6^A pattern distribution across the mRNA regions in brain, intestine and liver. m^6^A peaks were mapped back to the corresponding gene, and assigned as originated from 5′ UTR, coding region (CDS) or 3′ UTR. **d** Motif analysis of m^6^A peaks. Upper panel, GF tissues; lower panel, SPF tissues. **e** Venn diagram showing the differences of m^6^A peaks between GF and SPF samples. **f** Principal component analysis of input (IN) and IP samples. The label is for Sample_tissue_Seq, e.g., GF_B_IP stands for GF mouse, brain, m^6^A-IP. Tissue labels are: B, brain; I, intestine; L, liver. **g** Representative sequencing coverage of an mRNA in the brain showing a differential m^6^A peak in GF and SPF samples. **h** Transcript counts containing different m^6^A peak numbers in the brain. **i** m^6^A peak and exon density in the brain. **j** Abundance of m^6^A-containing transcripts in the brain. **k** mRNA m^6^A peak positions in the brain. **l** Reactome analysis of biological pathways of m^6^A-containing transcripts in the brain. **m** Venn diagram comparing the 4-week-old GF/SPF brain m^6^A peak-containing transcripts with those in the E13.5 embryonic brain. **n** Western blots of m^6^A writer proteins METTL3, METTL14, and eraser proteins FTO, ALKBH5 in the brain tissues. **o** Quantitation of m^6^A writer and eraser protein levels in the brain. Values are the means ± SD, *n* = 3, *P < 0.05, **P < 0.01, ***P < 0.001, Student’s *t*-test. **p** Quantitation of m^6^A writer and eraser protein levels in the intestine and liver. Values are the means ± SD, *n* = 3, *P < 0.05, Student’s *t*-test

We harvested three tissues of GF and SPF mice of the same genetic background at 4 weeks of age, brain, intestine, and liver, and performed m^6^A analysis in polyA-selected RNA by liquid chromatography/mass spectrometry (LC/MS) to determine the total m^6^A/A ratios and by the m^6^A-MeRIP sequencing to determine the transcriptomic m^6^A pattern and distribution. These three tissues were selected based on their pervasive studies in the literature on the GF and SPF mouse physiology. The m^6^A/A ratios of the polyA-selected RNA are in the expected range of 0.2%–0.6%; brain showed the highest m^6^A content for both GF and SPF mice, and brain and intestine showed higher m^6^A content in the GF mice (Fig. 1b). The polyA-selected RNA in kidney also showed higher m^6^A content in the GF mice (Supplementary information, Fig. S2a). The higher m^6^A content in the brain tissue was also observed in GF and SPF mice that were 10 weeks old (Supplementary information, Fig. S2b) and even 2 years old (Supplementary information, Fig. S2c). Our m^6^A-MeRIP results of all three tissues (Supplementary information, Table S1) showed the well-known m^6^A pattern across the mRNA transcripts such as the strong enrichment of m^6^A peaks at the junction of coding region (CDS) and 3′ UTR (Fig. 1c). We identified the m^6^A-containing transcripts that were present in all three GF or SPF mouse groups as “high confidence” data and used only these for further analysis (Supplementary information, Fig. S3). We recovered the known m^6^A installation consensus sequence, RRACH (R = A/G, H = A/C/U) among the m^6^A peaks with a preference of guanosine 5′ to the m^6^A site (Fig. 1d). We validated our sequencing results by quantitative RT-PCR of specific transcripts (Supplementary information, Fig. S4a). Our sequencing result was also consistent with the expected mRNA expression difference of GF versus SPF mouse reported in the literature^5,6^ (Supplementary information, Fig. S4b). These results validated the high-quality nature of our m^6^A-MeRIP data.

We identified several differences in m^6^A patterns between the GF and SPF tissues. First, the m^6^A peak distributions had distinct shapes among these tissues (Fig. 1c). When benchmarked against the m^6^A cluster near the stop codon, the GF brain m^6^A occurrence was higher in the CDS region compared to the SPF brain. Second, the m^6^A installation consensus sequences in GF and SPF tissues were deviated in brain, but identical in intestine and liver (Fig. 1d). Third, only 25% of the m^6^A peaks in the GF brain overlapped with those in SPF brain, whereas > 59% of the m^6^A peaks overlapped in GF and SPF intestine and liver (Fig. 1e). The large brain m^6^A peak differences was also shown by principal component analysis and their statistical significance in gene ontology analysis across all tissues (Fig. 1f and Supplementary information, Fig. S5), and was not derived from global transcript expression differences (Supplementary information, Fig. S6). On the other hand, SPF brain may have higher m^6^A modification fraction for some common m^6^A peaks (Supplementary information, Fig. S7). All together, these results indicate that the presence of microbiome has a profound influence on the cellular mRNA m^6^A patterns in a tissue-dependent manner. Alteration of the m^6^A pattern is most pronounced in the brain, where the m^6^A methylome is substantially reduced in the presence of microbiome.

We performed in-depth analysis for the GF and SPF brain tissues to further elucidate the m^6^A alteration in the mRNA (a representative read coverage plot shown in Fig. 1g). Among the 8643 and 2750 m^6^A-containing transcripts, 67 and 80% had only one m^6^A peak in the GF and SPF brains, respectively, and the GF/SPF transcript ratio was 2.6 (Fig. 1h). However, this GF/SPF ratio for the transcripts containing two or more m^6^A peaks steadily increased to 4.4 for two, 6.7 for three, and 9.2 for more than three m^6^A peaks. GF brain transcripts also had a broader distribution of m^6^A peak/exon ratios (Fig. 1i). These results indicate that more m^6^A clusters are present in individual GF than SPF brain transcripts. The abundance of the m^6^A-containing transcripts was lower in GF than SPF brain (Fig. 1j), which might be associated with a major known role of m^6^A in accelerating mRNA decay.^7^ More m^6^A peaks were present in all three mRNA regions in GF than SPF brain (Fig. 1k). The m^6^A location in different mRNA regions has been associated with different functions. For example, m^6^A in the 5′ UTR enhances translation through eIF3-dependent recruitment of the ribosome;^8^ m^6^A in the 3′ UTR regulates mRNA stability and translation efficiency that depend on the m^6^A reader proteins YTHDF1, YTHDF2 and YTHDF3; m^6^A in the CDS regulates splicing that involves the m^6^A reader proteins YTHDC1, hnRNPC and hnRNPG,^9,10^ and codon-dependent translational efficiency.^11^ The large increase of the m^6^A peaks in the GF brain therefore could affect the m^6^A-dependent mRNA function in several different ways. This multifaceted m^6^A effects in the brain were consistent with the reactome analysis of biological pathways that showed the top enriched categories for m^6^A-containing transcripts, including mRNA splicing, cell cycle and signaling (Fig. 1l).

The GF brain may represent an under-developed state due to the lack of its microbiome exposure.^12–14^ To obtain insight into whether this idea applies to m^6^A in the transcriptome, we compared our results with the published m^6^A patterns from the mouse embryonic brain^15^ using only the high-confidence m^6^A-containing transcripts from both studies (Fig. 1m). The E13.5 embryonic brain (7701) had a comparable amount of m^6^A-containing transcripts to the 4-week-old GF brain (8,643); the overlap between them was 60% for embryonic brain and 54% for GF brain. The embryonic brain (7701) had a much higher amount of m^6^A-containing transcripts than the 4-week-old SPF brain (2750); the overlap between them was 20% for embryonic brain and 57% for SPF brain. Ninety-seven percent of all GF and SPF brain m^6^A-containing transcripts overlapped, which explained the similar overlapping percentage of GF and SPF with the embryonic transcripts. These results suggest that in regards to m^6^A modification, the GF brain more closely resembles the embryonic brain than the SPF brain of the same age.

To obtain mechanistic understanding of m^6^A changes in GF and SPF tissues, we measured the levels of the mRNA m^6^A writer proteins METTL3 and METTL14, and the m^6^A eraser proteins FTO and ALKBH5 by western blot. We found that both m^6^A writer proteins and both m^6^A eraser proteins were highly overexpressed in the GF brain compared to the SPF brain (Figs. 1n, o, and Supplementary information, Fig. S8). In contrast to brain, the differential expression of these proteins in the intestine and liver was much less noticeable without a uniform trend (Fig. 1p). These results correlate well with the finding that the brain has the largest difference in the m^6^A pattern among the three tissues examined here. Furthermore, the simultaneous overexpression of the m^6^A writer complex and the erasers in the same tissue should increase the ability to rapidly tune the m^6^A pattern upon environmental changes.

In summary, here we show that the microbiome has a strong effect on host m^6^A mRNA modification. Among the brain, intestine and liver tissues, the largest effect is present in the brain, which is associated with overexpression of both m^6^A writer and eraser proteins; this result suggests that the brain tissue may be more sensitive to adjust the m^6^A methylome in response to the microbiome than other tissues. Future studies will reveal the specific microbial species and the molecular mechanisms that regulate the host m^6^A methylome.

## Supplementary information

Supplemental Information
