# Supplementary material for: Transcriptome-wide reprogramming of N6-methyladenosine modification by the mouse microbiome
Source: Cell Res. 2018 Dec 17;29(2):167–70. doi: 10.1038/s41422-018-0127-2 (PMC6355850; doi:10.1038/s41422-018-0127-2)

Page 1-6: Methods

Page 7: Table S1: Sequencing read counts and mapping rates.

Page 8: Supplemental Figure 1: Validation of germ-free mice and 16S rRNA amplicon sequencing of SPF mice.

Page 9: Supplemental Figure 2: Additional m^{6}A content analysis by LC/MS.

Page 10: Supplemental Figure 3: Venn diagram shows the overlap of m^{6}A peaks between GF and SPF brain samples.

Page 11: Supplemental Figure 4: Validation of sequencing results.

Page 12: Supplemental Figure 5: Gene ontology (GO) analysis of genes with diffPeak.

Page 13: Supplemental Figure 6: Expression analysis for transcripts that contain only m^{6}A peaks in the GF brain.

Page 14: Supplemental Figure 7: Sequencing coverage of an mRNA in the brain showing higher m^{6}A peak height in SPF than in GF samples.

Page 15: Supplemental Figure 8: Quantitation of m^{6}A writer METTL14 protein levels in the brain of 10-week-old mice.

Methods

Sample collection. Mouse tissues (brain, intestine, liver) dissected from SPF mice (4-week-old male C57BL/6 mice, n=3) and GF mice (4-week-old male C57BL/6 mice, n=3) were purchased from Taconic Biosciences. All GF and SPF mice in our study were fed with the same, autoclaved NIH-31M rodent diet. This diet has an open formula, which can be reviewed at https://www.taconic.com/quality/animal-diet/. Mouse feces samples were also collected to extract genomic DNAs. All tissues and mouse feces were freshly frozen on dry ice and stored at –80 °C.

Genomic DNA extraction, 16S rRNA gene PCR amplification. 200 mg mouse fecal pellets were suspended in 1 ml of sterile bacterial lysis buffer (50 mM Tris-HCl, pH 7.4, 400 mM NaCl, 100 mM EDTA, 0.5% SDS) with freshly added 20 µl of 20 mg/ml proteinase K (Roche). The stool mixtures were disrupted with glass beads (Sigma) using beads beaters followed by incubation at 55 °C overnight. The homogenized mixture was centrifuged at 16,000× *g* for 15 min and 600 µl supernatant was transferred to a new 1.5 ml microfuge tube. Following the

addition of 200 µl of 5 M NaCl to the fecal mixture and incubation on ice for 10 min, the samples were centrifuged at 16,000× *g* for 15 min. Fecal DNAs were extracted from the supernatants by phenol/chloroform/isoamyl alcohol (25:24:1) followed by ethanol precipitation. The genomic DNA was resuspended in 100 µl nuclease free water, the concentration of each DNA extract was determined spectrophotometrically at 260 nm using a NanoDrop 2000 Spectrophotometer (Thermo Scientific) and stored at –80 °C until used. The condition of GF mice was confirmed by 16S rRNA PCR amplification using two sets of universal bacterial primers (27F: 5'-AGAGTTTGATCCTGGCTCAG-3', 1492R: 5'-TACCTTGTTACGACTT-3'; 8F: 5'-AGAGTTTGATCCTGGCTCAG-3', 1541R: 5'-AAGGAGGTGATCCAGCCGCA-3'). 20 ng of sample DNA from GF mouse or SPF mouse was used in PCR reaction using Phusion High-Fidelity DNA Polymerase (Thermo Scientific). PCR products were visualized on a 1% agarose gel stained with ethidium bromide under UV light to confirm the absence and presence of the 1464 bp and 1533 bp bands.

16S rRNA gene amplicon sequencing. Genomic DNA samples from SPF mice were sent to Marine Biological Laboratory for 16S rRNA sequencing to identify genus-level taxonomy. For 16S sequencing we produced PCR amplicon libraries targeting the V4 region of the 16S rRNA encoding gene (515F-806R) in the metagenomic DNA using a barcoded primer set adapted for the Illumina HiSeq2000 and MiSeq. Illumina MiSeq sequencing at the Environmental Sample Preparation and Sequencing Facility (ESPSF) at Marine Biological Laboratory generated 151bp paired-end amplicon sequences. We removed low-quality reads from the raw sequencing results using illumina-utils [1] (available from https://github.com/merenlab/illumina-utils). We then used the program 'iu-merge-pairs' with default parameters, which merged partially overlapping paired-end Illumina reads while simultaneously removing any pair with more than 3 mismatches at the overlapped region. For pairs with fewer mismatches, we picked the base to be used in the final merged sequence from the read with the higher Q-score. We used Minimum Entropy Decomposition [2] with default parameters to cluster high-quality 16S rRNA gene amplicons at single base resolution, and GAST [3] to assign taxonomy to each read individually.

RNA isolation and purification. Tissues were homogenized in 2 ml TRIzol with glass beads using homogenizer. Total RNA was isolated from frozen tissues using TRIzol reagent (Ambion)

following the manufacturer's protocol. RNA samples were dissolved in RNase-free water. The concentration and purity of total RNA in each sample were quantified spectrophotometrically at 260 nm using a NanoDrop 2000 Spectrophotometer (Thermo Scientific). RNA samples were kept at –80 °C until used. To obtain mRNA, total RNA was purified using PolyATtract® mRNA Isolation System IV (Promega) followed by RiboMinus transcriptome isolation kit for human/mouse (Invitrogen).

LC-MS/MS quantification of m^6A mRNA modification. 150 ng purified mRNAs were digested by 1 U nuclease P1 (Sigma, N8630) in 20 µl of buffer containing 25 mM NaCl and 2.5 mM $ZnCl_2$ for 2 h at 42 °C, followed by the addition of NH_4HCO_3 (1 M, 3 µl) and alkaline phosphatase (1 U, Sigma, P5931) and incubation at 37 °C for 2 h. The samples were then filtered (0.22 µm, Millipore) and injected into a C18 reverse phase column coupled online to Agilent 6460 LC-MS/MS spectrometer in positive electrospray ionization mode. The nucleosides were quantified by using retention time and the nucleoside to base ion mass transitions (268-to-136 for A; 282-to-150 for m^6A). Quantification was performed by comparing with the standard curve obtained from pure nucleoside standards running with the same batch of samples. The ratio of m^6A/A was calculated based on the calibrated concentrations.

MeRIP-seq/m^6A-seq. MeRIP experiments were carried out according to previously published protocols [4,5]. Briefly, polyA selection and ribodepletion-treated mRNA samples (1 µg of each) from tissues (brain, intestine, liver) were used for RNA fragmentation followed by immunoprecipitation with EpiMark® N6-Methyladenosine Enrichment Kit (New England Biolabs, E1610S). RNAs were eluted from the protein G magnetic beads in 30 µl of Buffer RLT (Qiagen, 79216) followed by precipitation in 100% ethanol. RNAs were dissolved in 12 µl RNase-free water. 120 ng of input RNA and immunoprecipitated RNAs were used for library preparation using Illumina TruSeq Stranded mRNA Library Preparation Kit (Illumina) according to the manufacturer's instructions. The concentration and quality of libraries were checked by the Agilent 2100 bioanalyzer. RNA sequencing was carried out at the University of Chicago Genomics Facility on an Illumina HiSeq2500 platform to generate 100-bp paired-end reads.

m^{6}A-seq data analyses.

(i) *RNA-seq analysis:* MeRIP-seq reads of each sample were aligned to the mouse reference genome (mm10) using HISAT2 (version 2.1.0) software. For the paired-end 100 nt sequences, default parameter was set for strand-specific mapping mode. The differential expression analysis was based on data from input sample, which can be treated as a traditional RNA-seq dataset. Quantification of the expression of protein-coding genes was calculated by FeatureCount (version 1.6.0) with default parameters. DESeq2 and edgeR package were applied to identify differentially expressed genes, then their common results were considered as the high-confidential DEgenes. Q-value ≤ 0.05 and $\log_2$fold change ≥ 1 were set as the thresholds of significance.

(ii) m^{6}A peak calling: m^{6}A peaks were identified by comparing the reads count between IP and input data in each continuous region in transcript. Generally, mapping results from IP and input were fed into the R package exomePeak (version 2.13.2) to identify the regions enriched of reads from IP sample comparing to input sample. A graphical model-based method is used to calculate the enrichment score, and then normalized to the sequencing depth. Default parameters of exomePeak were used and cutoff *P*-value was set to 0.05. The differentially methylated m^{6}A peaks (diffPeaks) between GF and SPF sample were identified by the R package MeTDiff. *P*-value was set to 0.05 as the threshold of significance.

The algorism we used above to identify m^{6}A peaks considered all 3 replicates for the input and IP samples simultaneously. m^{6}A peaks were called only for the significant peaks that passed our statistical definition above. An alternative method is to first consider the input and IP sequencing data for each sample separately, followed by overlapping the three samples to obtain common m^{6}A peaks. We also performed this alternative analysis; the result for the brain samples is shown in Fig. S3. In this case, the GF samples showed a 3.6-fold higher number of m^{6}A peaks than the SPF samples, which is very similar to the GF/SPF m^{6}A peak ratio of 3.8 in the first analysis method (Fig. 1e).

(iii) m^{6}A motif search: Bedtools(version 2.26.0) was used to get the sequences of the peak regions; then the Dreme (from the software suite Homer, version 4.12.0) algorism was used to search for the sequence motif which concurrently appeared in multiple regions; the ratio above random distributions was calculated.

(iv) Metagene plot: The metagene plot exhibits the m^6A enrichment on mRNA. The midpoint position of each peak was extracted to represent the m^6A site; then the m^6A sites were mapped to transcripts, and their relative distances to the 5'UTR, CDS, and 3'UTR region of the mRNA were calculated. The cumulated densities of m^6A sites belonging to each category were plot by a curve with smoothing process.
(v) Gene ontology and Reactome analysis: Functional annotation and enrichment analysis were performed by KOBAS (version 3.0). First, specific gene list such as DEgenes or m^6A containing genes were generated from other procedures; then the corresponding gene IDs were mapped to Gene Ontology/Reactome Pathway databases. P-value or a probability of enrichment and enrichment score were calculated based on results of multiple statistic methods. P-value < 0.05 was set as the cutoff to obtain significant enrichment results.
(vi) Pairwise comparison of the m^6A atlas in GF mouse brain to prenatal mouse: The m^6A epitranscriptome data was downloaded from public database (GSE99017)[6]. The transcripts containing m^6A peaks were cross-compared between each two samples (GF vs SPF, GF vs Prenatal, SPF vs Prenatal), and the overlap peak counts and specific peak counts in each sample were depicted by Venn diagrams.

Real-time RT-PCR. The iScript™ cDNA Synthesis Kit (Bio-Rad) was used to synthesize cDNA with 1 μg of brain total RNA. The cDNAs were 20-fold diluted for real-time RT-PCR reaction using iTaq Universal SYBR Green Supermix (Bio-Rad). The mouse housekeeping gene actin was used as internal control. The fluorescence intensity of the amplification process was monitored using CFX96 Touch™ Real-Time PCR Detection System (Bio-Rad). Primers used for RT-qPCR are the following: Csnk1g2-F, CAAACTTCCGAGTCGGCAAGA, Csnk1g2-R, GCTTGTAAAAGCGGTACTCCAG; Kcnc2-F, TCGCCACCCAGGAGTATTC, Kcnc2-R, CTCCACGTCGGTCTCATCG; Scaf8-F, GATATGGCGGCAGGGATTCC, Scaf8-R, AGGACTTTGTAGGATCTGAGCA; Zic1-F, CAGTATCCCGCGATTGGTGT, Zic1-R, GCGAACTGGGGTTGAGCTT; Rbm15-F, CGAGTCCGCTGTGTGAAAC, Rbm15-R, TCCCCACGAGAACTGGAGTC; Actin-F, GGCTGTATTCCCCTCCATCG, Actin-R, CCAGTTGGTAACAATGCCATGT.

Western Blotting. About 50 mg of frozen tissue was homogenized using tissue homogenizer in 500 µl RIPA buffer (Thermo Fisher Scientific) with freshly added 1 mM phenylmethylsulfonyl fluoride. The resulting suspension was centrifuged for 30 min at 15,000× *g*, the supernatant was collected for Western Blotting analysis and protein concentrations were measured by BCA assays. The samples were subjected to polyacrylamide gel electrophoresis and transferred onto a polyvinylidene difluoride membrane. Primary antibodies used in this study include rabbit anti-METTL3 antibody (ab195352, Abcam, 1: 1000), rabbit anti-METTL14 antibody (HPA038002, Sigma, 1:3000), mouse anti-FTO antibody (ab92821, Abcam, 1:1000), rabbit anti-ALKBH5 antibody (HPA007196, Sigma, 1:1000), mouse anti-beta actin antibody (ab6276, Abcam, 1:6000). Proteins were detected using Amersham™ ECL™ Western Blotting Detection Reagents (GE Healthcare). Intensities of the protein bands were quantified using the Image lab Software.

Statistical Analysis. Data are presented as mean ± SEM. Asterisks (*) represent significant differences between SPF mice and GF mice, as determined by the Student's *t*-test ($P < 0.05$).

Data availability: The RNA-seq and m^{6}A-seq data generated by this study have been deposited in the NCBI GEO database under the accession number GSE120262.

References

1 Eren, A. M., Vineis, J. H., Morrison, H. G. & Sogin, M. L. A filtering method to generate high quality short reads using illumina paired-end technology. *PLoS One* **8**, e66643 (2013).

2 Eren, A. M. *et al.* Minimum entropy decomposition: unsupervised oligotyping for sensitive partitioning of high-throughput marker gene sequences. *ISME J* **9**, 968-979 (2015).

3 Huse, S. M. *et al.* Exploring microbial diversity and taxonomy using SSU rRNA hypervariable tag sequencing. *PLoS Genet* **4**, e1000255 (2008).

4 Dominissini, D. *et al.* Topology of the human and mouse m6A RNA methylomes revealed by m6A-seq. *Nature* **485**, 201-206 (2012).

5 Meyer, K. D. *et al.* Comprehensive analysis of mRNA methylation reveals enrichment in 3' UTRs and near stop codons. *Cell* **149**, 1635-1646 (2012).

6 Yoon, K. J. *et al.* Temporal Control of Mammalian Cortical Neurogenesis by m(6)A Methylation. *Cell* **171**, 877-889 (2017).

7 Selwyn, F. P., Cui, J. Y. & Klaassen, C. D. RNA-Seq Quantification of Hepatic Drug Processing Genes in Germ-Free Mice. *Drug Metab Dispos* **43**, 1572-1580 (2015).

8 Pan, W. H. *et al.* Exposure to the gut microbiota drives distinct methylome and transcriptome changes in intestinal epithelial cells during postnatal development. *Genome Med* **10**, 27 (2018).

Table S1: Sequencing read counts and mapping rates.

Sample	Tissue	Mouse	Library	Total Reads	Mapped reads	Mapped rate
Liver_GF_IP	Liver	GF	IP	58045184	54289661	0.94
	Liver	GF	IP	88294674	67395325	0.76
	Liver	GF	IP	43251510	41041358	0.95
Liver_SPF_IP	Liver	SPF	IP	49214586	46867050	0.95
	Liver	SPF	IP	47773932	42303817	0.89
	Liver	SPF	IP	45944396	43909059	0.96
Liver_GF_Input	Liver	GF	Input	57918010	54674601	0.94
	Liver	GF	Input	68552738	56679404	0.83
	Liver	GF	Input	52172926	49746885	0.95
Liver_SPF_Input	Liver	SPF	Input	62500400	59625382	0.95
	Liver	SPF	Input	57455828	52606556	0.92
	Liver	SPF	Input	68409054	65419578	0.96
Intestine_GF_IP	Intestine	GF	IP	45036616	32588495	0.72
	Intestine	GF	IP	66993826	60917486	0.91
	Intestine	GF	IP	39581896	32825266	0.83
Intestine_SPF_IP	Intestine	SPF	IP	44822120	34235135	0.76
	Intestine	SPF	IP	40342562	31168663	0.77
	Intestine	SPF	IP	54988262	36050305	0.66
Intestine_GF_Input	Intestine	GF	Input	63500256	52057510	0.82
	Intestine	GF	Input	83686008	72221025	0.86
	Intestine	GF	Input	75925698	64688695	0.85
Intestine_SPF_Input	Intestine	SPF	Input	72036820	56966717	0.79
	Intestine	SPF	Input	82160556	60659138	0.74
	Intestine	SPF	Input	82346178	64345303	0.78
Brain_GF_IP	Brain	GF	IP	77135736	59996175	0.78
	Brain	GF	IP	62295272	55293283	0.89
	Brain	GF	IP	35582488	29722052	0.84
Brain_SPF_IP	Brain	SPF	IP	66400994	56288123	0.85
	Brain	SPF	IP	43745686	37393812	0.85
	Brain	SPF	IP	81147972	41921042	0.52
Brain_GF_Input	Brain	GF	Input	77495026	73558279	0.95
	Brain	GF	Input	91194956	87045586	0.95
	Brain	GF	Input	60717946	57542397	0.95
Brain_SPF_Input	Brain	SPF	Input	67481506	62845527	0.93
	Brain	SPF	Input	62798580	55400907	0.88
	Brain	SPF	Input	55048168	48618542	0.88

Supplemental Figure 1: Validation of germ-free mice and 16S rRNA amplicon sequencing of SPF mice. (a) The condition of GF mice was confirmed by 16S rRNA PCR amplification targeting universal bacterial rRNA sequences in the DNA extracts from the intestinal content. Two sets of universal bacterial primers were used (see Methods). PCR products from GF (n=3) and SPF (n=3) were visualized on a 1% agarose gel stained with ethidium bromide under UV light to confirm the absence and presence of the 1464 bp and 1533 bp bands. (b) 16S rRNA gene amplicon sequencing identified genus-level taxonomy in the SPF mouse stools.

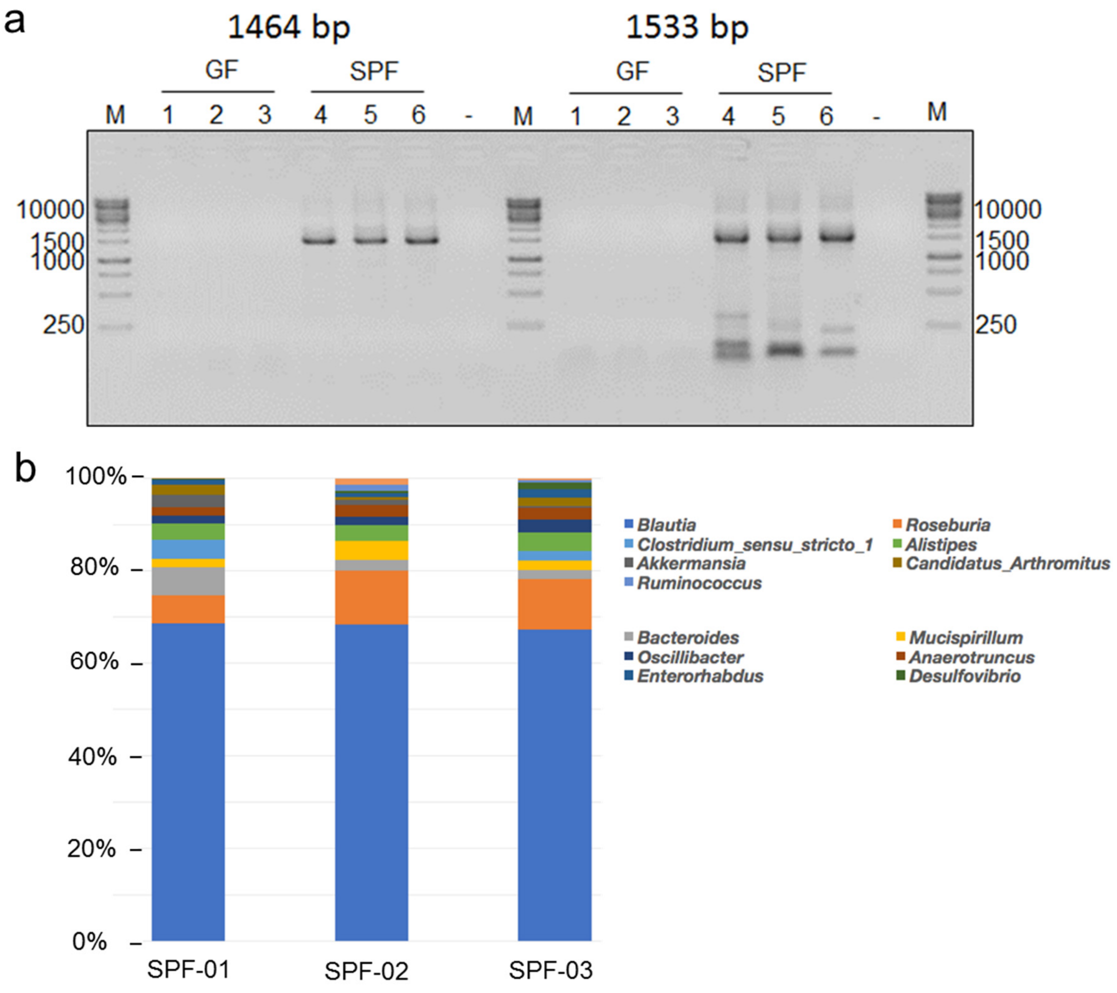

Supplemental Figure 2: Additional m^{6}A content analysis by LC/MS. (a) Total m^{6}A/A ratio of polyA-selected and ribo-minus treated RNA from kidney of the same GF and SPF mouse group in Figure 1. Values are the means ± standard deviation (SD), n = 3, *$P < 0.05$, Student's *t*-test. (b) Total m^{6}A/A ratio of polyA-selected and ribo-minus treated RNA from brain of 10-week-old mice, and of 2-year-old mice (c).

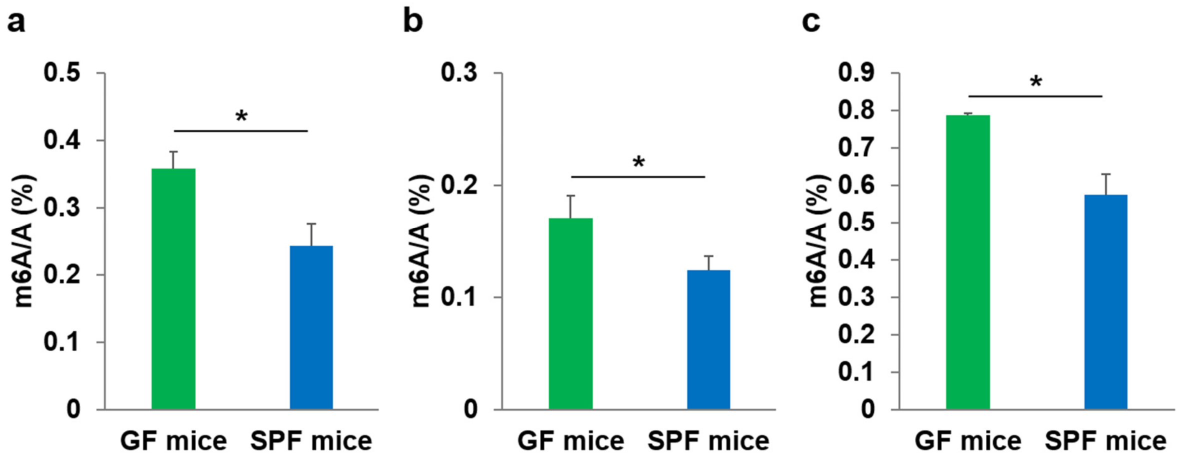

Supplemental Figure 3: Venn diagram showing the overlap of m^{6}A peaks between GF and SPF brain samples. The algorism we used to identify m^{6}A peaks in Fig. 1e considered all 3 replicates for the input and IP samples simultaneously. Shown here is the m^{6}A peak overlap using another method that first considers the input and IP data for each sample separately, followed by overlapping the three samples to obtain common m^{6}A peaks. The number of common m^{6}A peaks according to the simultaneous method was 13,323 for GF and 3,502 for SPF mice (Fig. 1e). The number of common m^{6}A peaks according to the individual sample method was 12,054 for GF and 3,368 for SPF mice as shown here.

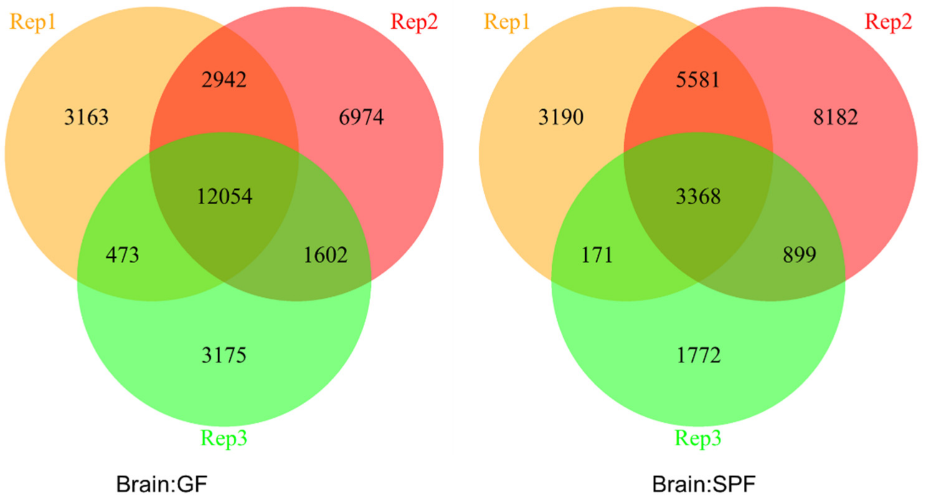

Supplemental Figure 4: Validation of sequencing results. (a) qRT-PCR of five mRNA transcripts from the brain samples. Values are the means ± standard deviation (SD), n = 3, **P* < 0.05, ***P* < 0.01, Student's *t*-test. (b) mRNA expression data from our sequencing results of previously characterized transcripts in the literature that faithfully reported the germ-free nature of our samples. The selected liver genes are from [7], and intestine genes are from [8].

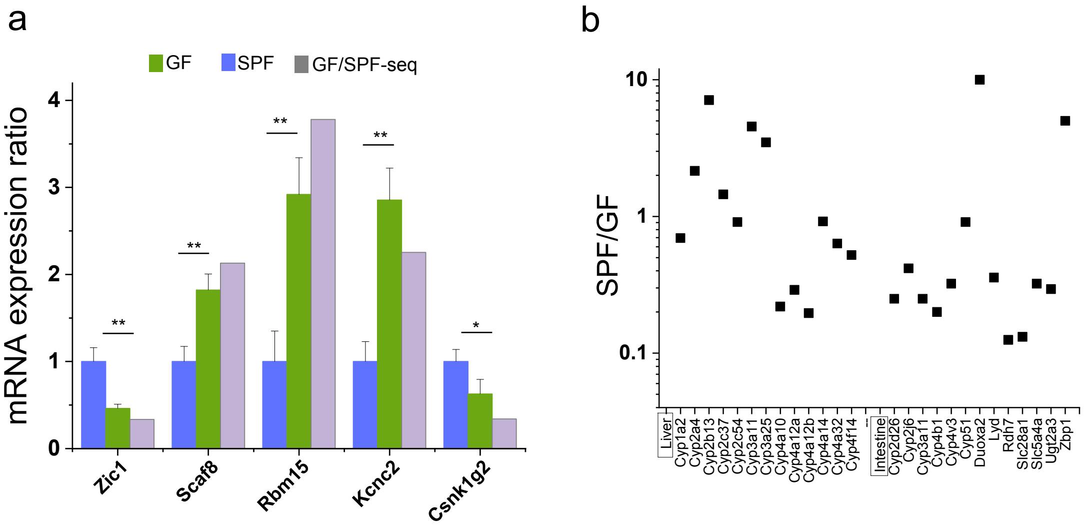

Supplemental Figure 5: Gene ontology (GO) analysis of genes with diffPeak. The diffPeak identifies differentially methylated m^{6}A peaks, which was calculated by the R package MeTDiff. The score represents the enrichment of each GO term by statistical calculation.

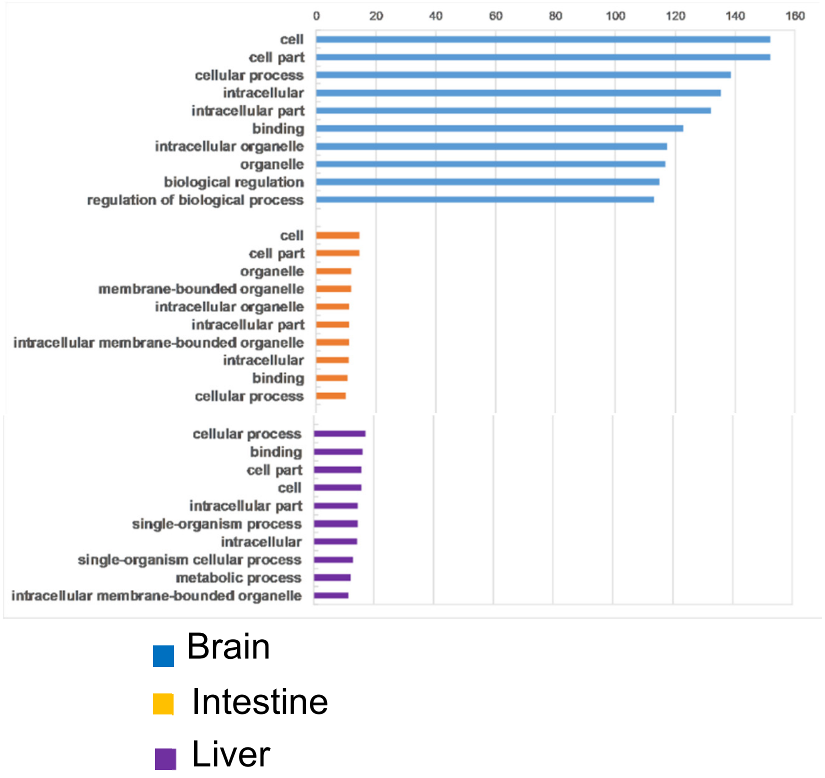

Supplemental Figure 6: Expression analysis for transcripts that contain only m^6A peaks in the GF brain. mRNA expression analysis was performed using the input (non-IP) sequencing data for the > 9,000 transcripts containing m^6A peaks in the GF but not in the SPF samples. Our differential methylated peak analysis already considered and normalized the transcript expression levels.

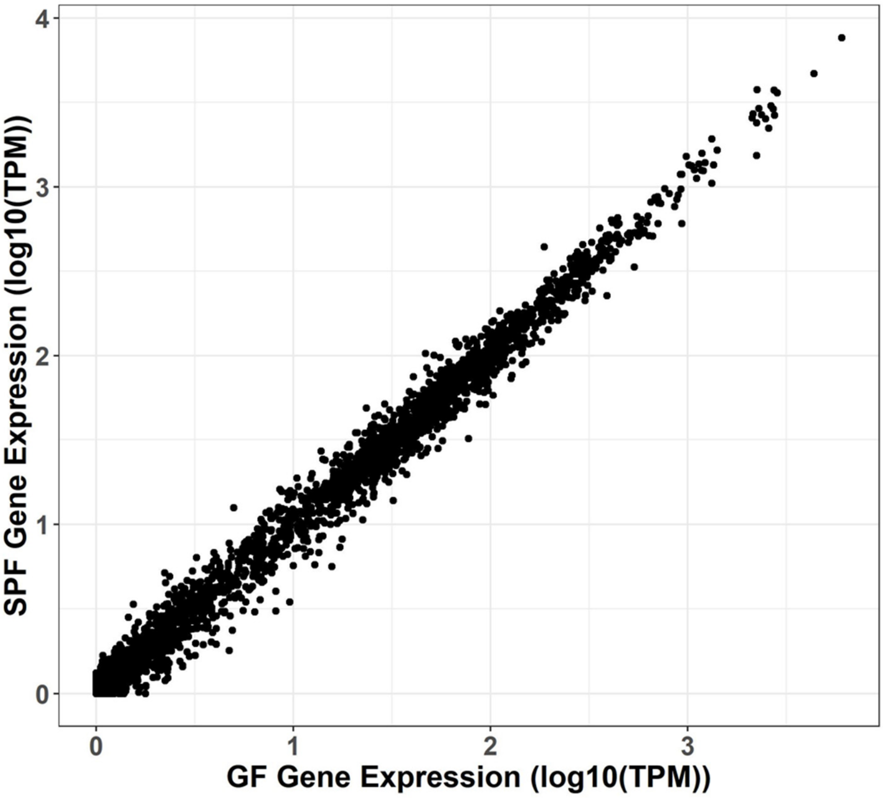

Supplemental Figure 7: Sequencing coverage of two mRNA examples in the brain showing higher m^{6}A peak height in SPF than in GF samples.

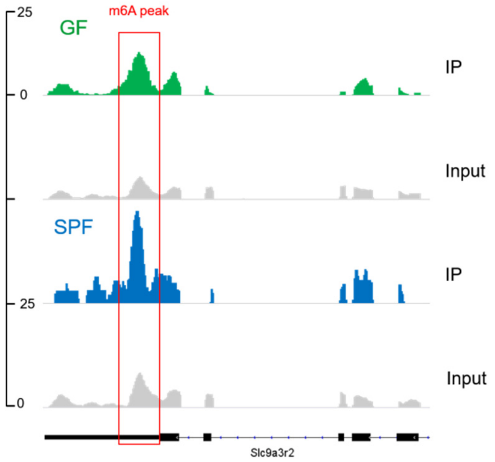

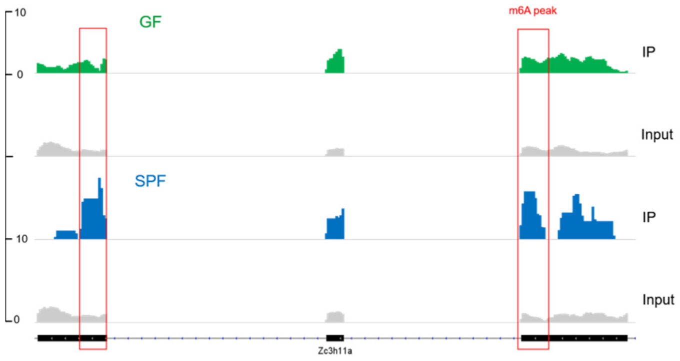

Supplemental Figure 8: Quantitation of m^{6}A writer METTL14 protein levels in the brain of 10-week-old mice. Values are the means ± standard deviation (SD), n = 3, *$P < 0.05$, Student's *t*-test.

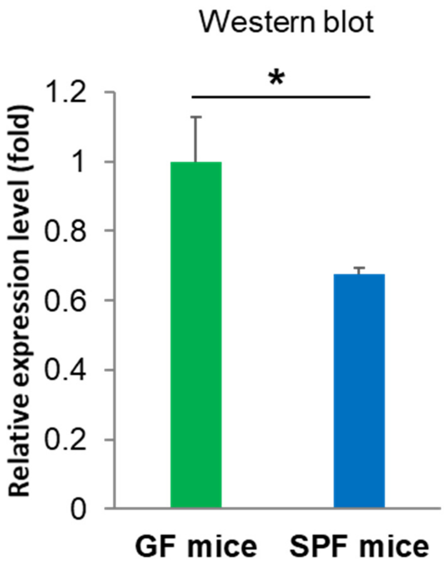

Supplement: Supplementary file 1 — Supplemental Information [file 41422_2018_127_MOESM1_ESM.pdf]
